# 3D Echocardiography for Rheumatic Heart Disease Analysis: Ready for Prime Time

**DOI:** 10.3389/fcvm.2021.676938

**Published:** 2021-07-20

**Authors:** Marcelo Luiz Campos Vieira, Carlos Eduardo de Barros Branco, Alessandra Santos Lima Gazola, Paulo Pinto Alves Campos Vieira, Luiz Alberto Benvenuti, Léa Maria Macruz Ferreira Demarchi, Paulo Sampaio Gutierrez, Vera Demarchi Aiello, Flávio Tarasoutchi, Roney Orismar Sampaio

**Affiliations:** ^1^Hospital Israelita Albert Einstein, São Paulo, Brazil; ^2^Heart Institute (InCor), São Paulo University Medical School, São Paulo, Brazil; ^3^Hospital 9 de Julho, São Paulo, Brazil; ^4^Santo Amaro University Medical School, São Paulo, Brazil

**Keywords:** rheumatic disease, echocardiography, valvulopathy, three dimensional echocardiography, diagnosis

## Abstract

Rheumatic heart disease (RHD) remains to be a very important health issue worldwide, mainly in underdeveloped countries. It continues to be a leading cause of morbidity and mortality throughout developing countries. RHD is a delayed non-suppurative immunologically mediated inflammatory response to the throat infection caused by a hemolytic streptococcus from the A group (*Streptococcus pyogenes*). RHD keeps position 1 as the most common cardiovascular disease in young people aged <25 years considering all the continents. The disease can lead to valvular cardiac lesions as well as to carditis. Rheumatic fever valvular injuries lead most commonly to the fusion and thickening of the edges of the cusps and to the fusion, thickening, and shortening of the chordae and ultimately to calcification of the valves. Valvular commissures can also be deeply compromised, leading to severe stenosis. Atrial and ventricular remodeling is also common following rheumatic infection. Mixed valvular lesions are more common than isolated valvular disorders. Echocardiography is the most relevant imaging technique not only to provide diagnostic information but also to enable prognostic data. Further, it presents a very important role for the correction of complications after surgical repair of rheumatic heart valvulopathies. Three-dimensional (3D) echocardiography provides additional anatomical and morphofunctional information of utmost importance for patients presenting rheumatic valvopathies. Accordingly, three-dimensional echocardiography is ready for routine use in patients with RHD presenting with valvular abnormalities.

## Introduction

### Epidemiology of Rheumatic Heart Disease: Distribution in the US and Worldwide

#### Brief Clinical Features and Pathogenesis

Rheumatic fever (RF) and rheumatic heart disease (RHD) continue to be very important health issues across different continents, affecting mainly developing or average income countries, as determined by the World Heart Federation position statement and by the Health Statistics Census from those nations (1, 2). Globally, RHD is the most common cardiovascular disease in young people aged <25 years (1). Therefore, RHD continues to be a very important cause of morbidity and mortality in developing countries. This scenario is very different and diverse in high-income countries. In such countries, RHD has mostly been eradicated, but a new burden of disease is possible due to migration flow (1). Another problem is that RHD may be under-observed and underdiagnosed in developing countries due to the scarcity of related studies, very small private and public investments, and the need for comprehensive register-based control programs (1–4). RF caused by Group A streptococcus/hemolytic streptococcus (GAS) (*Streptococcus pyogenes*) can be observed in cases of throat infection after tonsillitis in high-income countries. However, such infections in developing or average-income countries can lead to carditis and permanent valve damage as a consequence of repeated attacks of RF (1, 3). Worldwide, RF or RHD may be the cause of death in 233,000–500,000 patients per year (2). In 2015, an estimated 319,400 deaths occurred due to RHD from 33.4 million cases across different continents, and 10.5 million disability-adjusted life-years were lost due to RHD (5).

Clinical manifestations of acute RF can be pleomorphic, including fever, arthritis, carditis, chorea, subcutaneous nodules, and erythema marginatum. The prevalence rates of RHD in school-age children in developing countries vary from one to five cases per 1,000, mainly in sub-Saharan Africa (6). In some African and Asian countries, the rate of subclinical carditis due to RF has been better understood with the use of portable echocardiography for disease screening in schoolchildren, which provides additional diagnostic information for clinical evaluation (6, 7). Mitral valve involvement was observed in most cases (6). After the use of echocardiographic screening protocols, the prevalence of RHD was considerably higher (10 times) (8). Another important issue is that RHD prevalence may be higher in children over 15 years of age, as suggested by data from Australian aborigines (7, 8). Evidence of subclinical RHD is crucial since patients can develop chronic valvular disease. The World Heart Federation standardized echocardiographic criteria for rheumatic carditis in 2012, using three different categories (definite RHD, borderline RHD, and normal), based on 2D, continuous-wave, and color-Doppler echocardiography (9, 10).

It is known that ~60% of RHD patients who present with heart failure during their first acute RF attack demonstrate valvular disease 10 years later (11, 12). Prognostic factors are highly dependent on the occurrence of carditis and the recurrent attack rate. It is well-understood that the burden of RHD has decreased globally, despite the significant rates throughout the poorer regions of the Earth.

The pathogenesis of acute rheumatic carditis seems to be related to the immune-mediated response following GAS infection, the production of antibodies against GAS pharyngitis, and GAS production of aggressive elements such as streptococcal M protein, which can trigger an autoimmune response.

The activation and infiltration of T cells into the valves produce a disarray of the normal valvar cell arrangement and changes in valvar collagen. There is also activation of inflammatory cytokines, granulomatous inflammation, and CD4+ T cell-mediated delayed hypersensitivity against GAS. The pathognomonic lesions of the rheumatic process in the endocardium of the heart valve and in the myocardium are called Aschoff bodies and are observed as granulomatous inflammation. Valve lesions may be progressive, leading to chronic sequelae such as valvular stenosis and insufficiency, as well as to carditis, heart failure, and pulmonary hypertension. In addition, these aggressive effects depend on evidence of a susceptible host, as well as the level of host autoimmune response.

## Tridimensional Echocardiography

Echocardiography, in its various modalities, is a diagnostic imaging investigation technique that has clinical applications in countless scenarios for various heart diseases (13–48).

From the first observations in M-mode, echocardiography has had several advances in technique with the advent of two-dimensional (2D) transthoracic echocardiography with the Doppler technique (and its various presentations), transesophageal echocardiography, strain from speckle-tracking technique and echocardiography, and 3D transthoracic and transesophageal echocardiography with pocket transducers and cell phone acquisition using nanotechnology and miniaturization (Figures 1–15).

**Figure 1:**
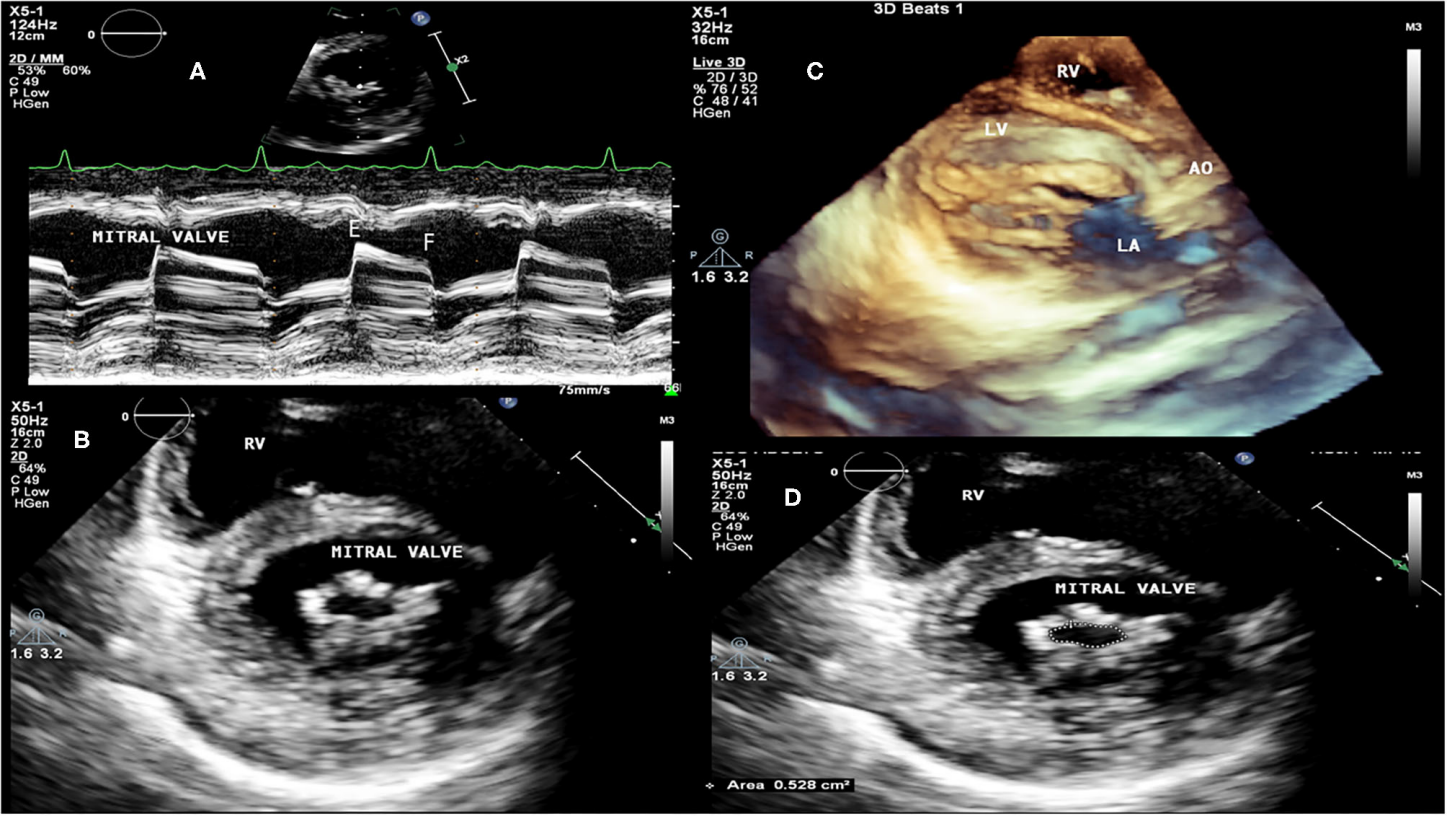
RHD (mitral valve stenosis). **(A)** M mode transthoracic echocardiography. Demonstration of patient with severe mitral valve stenosis (rectification of E to F slope), presenting atrial fibrillation. **(B)** 2D transthoracic echocardiography, transversal view, thickening of mitral valvular leaflets, “buttonhole” shape of the mitral valve. **(C)** 3D transthoracic echocardiography, mitral valve chordae fusion. **(D)** 2D transthoracic echocardiography, transversal view, measurement of the mitral valve area by planimetry: 0.528 cm^2^. RV, right ventricle; LV, left ventricle; AO, aorta; LA, left atrium.

**Figure 2:**
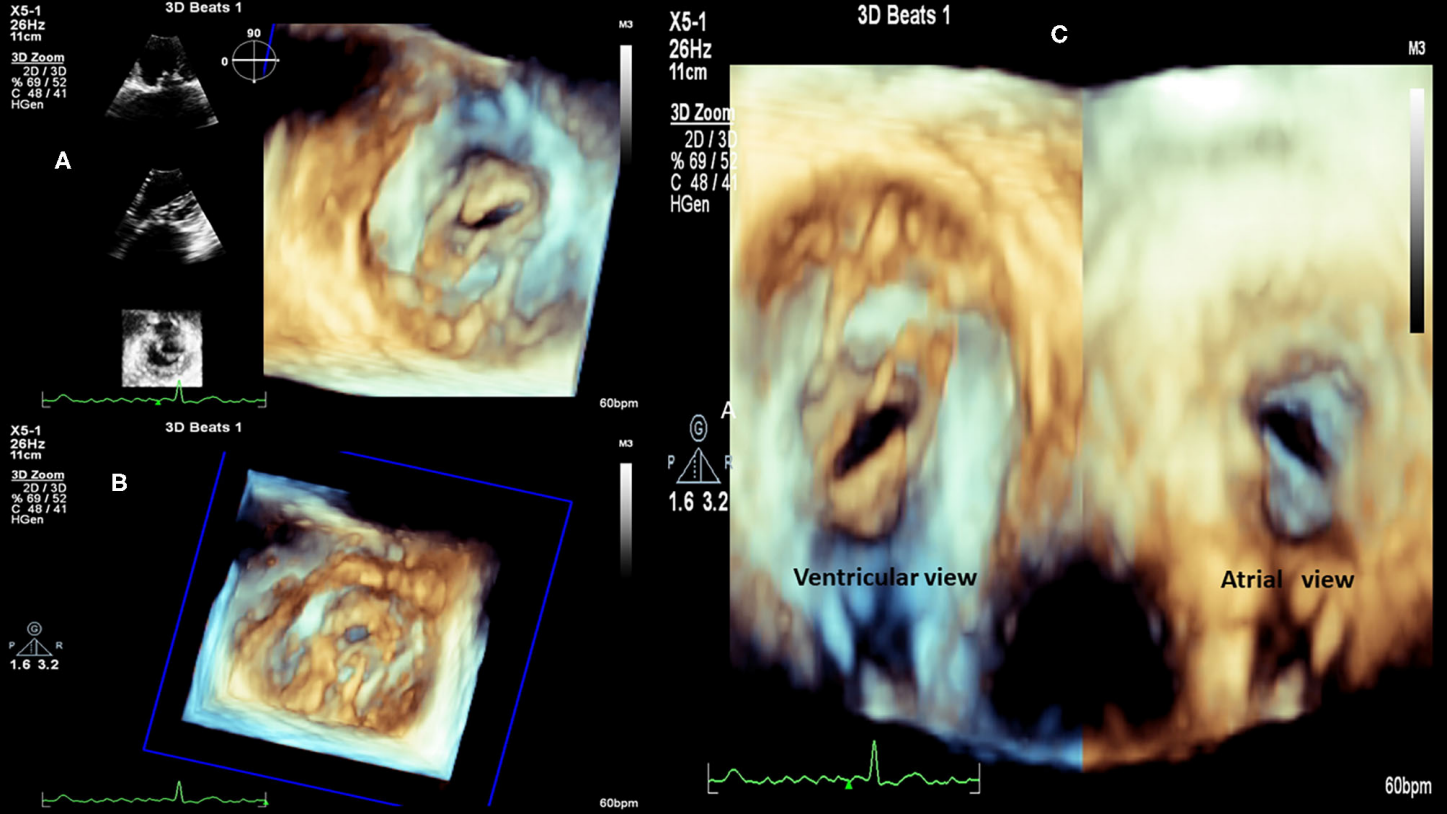
RHD (Mitral valve stenosis). **(A)** 3D transthoracic echocardiography, left atrium view, 2D reference structures, patient with severe valve mitral stenosis. **(B)** 3D transthoracic echocardiography, patient with severe valve mitral stenosis. **(C)** 3D transthoracic echocardiography, view from left atrium (right) and from the left ventricle (left), patient with severe valve mitral stenosis.

**Figure 3:**
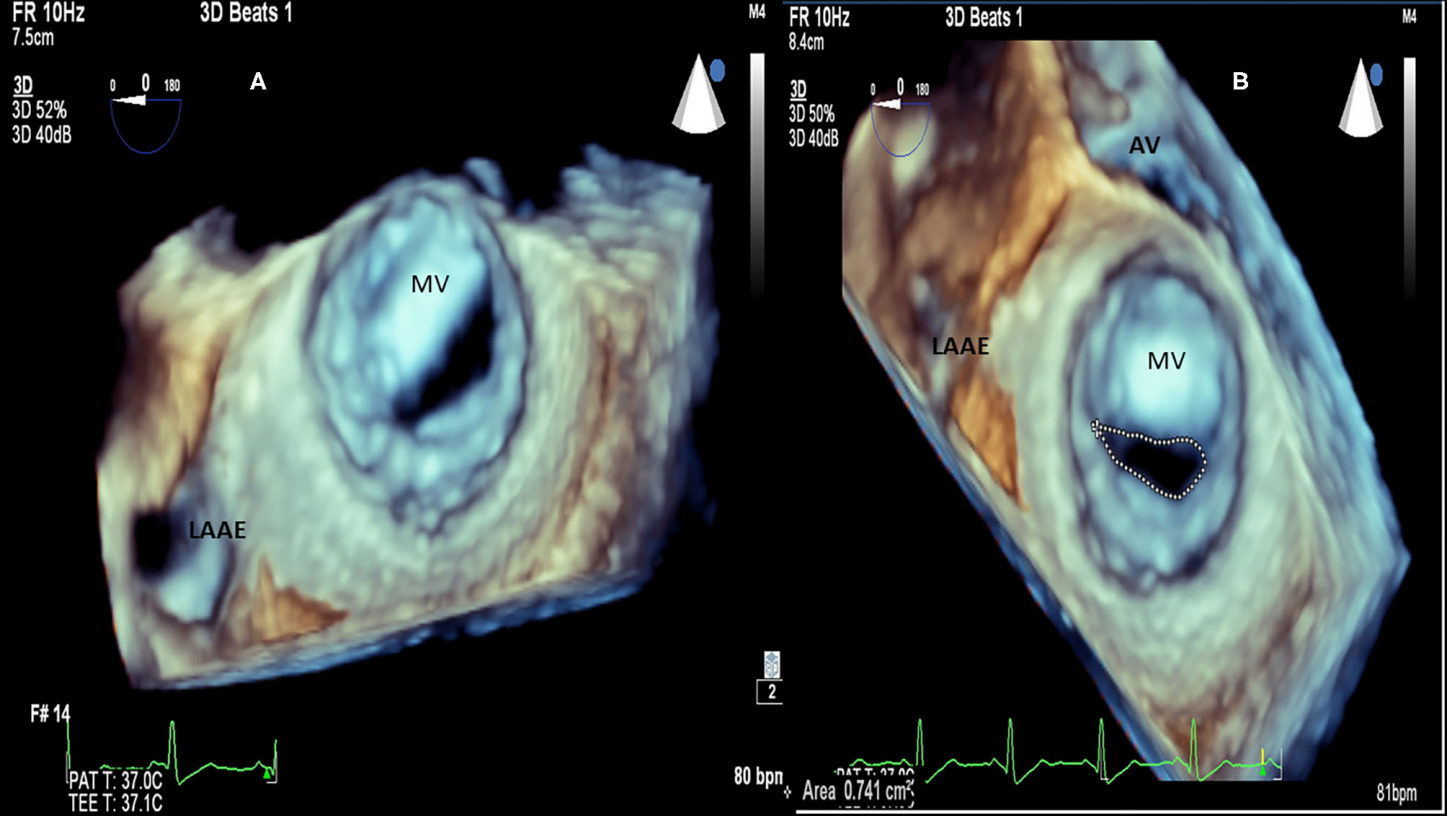
RHD (Mitral valve stenosis). **(A)** 3D transesophageal echocardiography. Demonstration of patient with severe mitral valve stenosis, “fishmouth” shape of the mitral valve. **(B)** 3D transesophageal echocardiography, same patient, measurement of the mitral valve area by planimetry: 0.741 cm^2^. LAAE, left atrium appendage; AV, aortic valve; MV, mitral valve.

**Figure 4:**
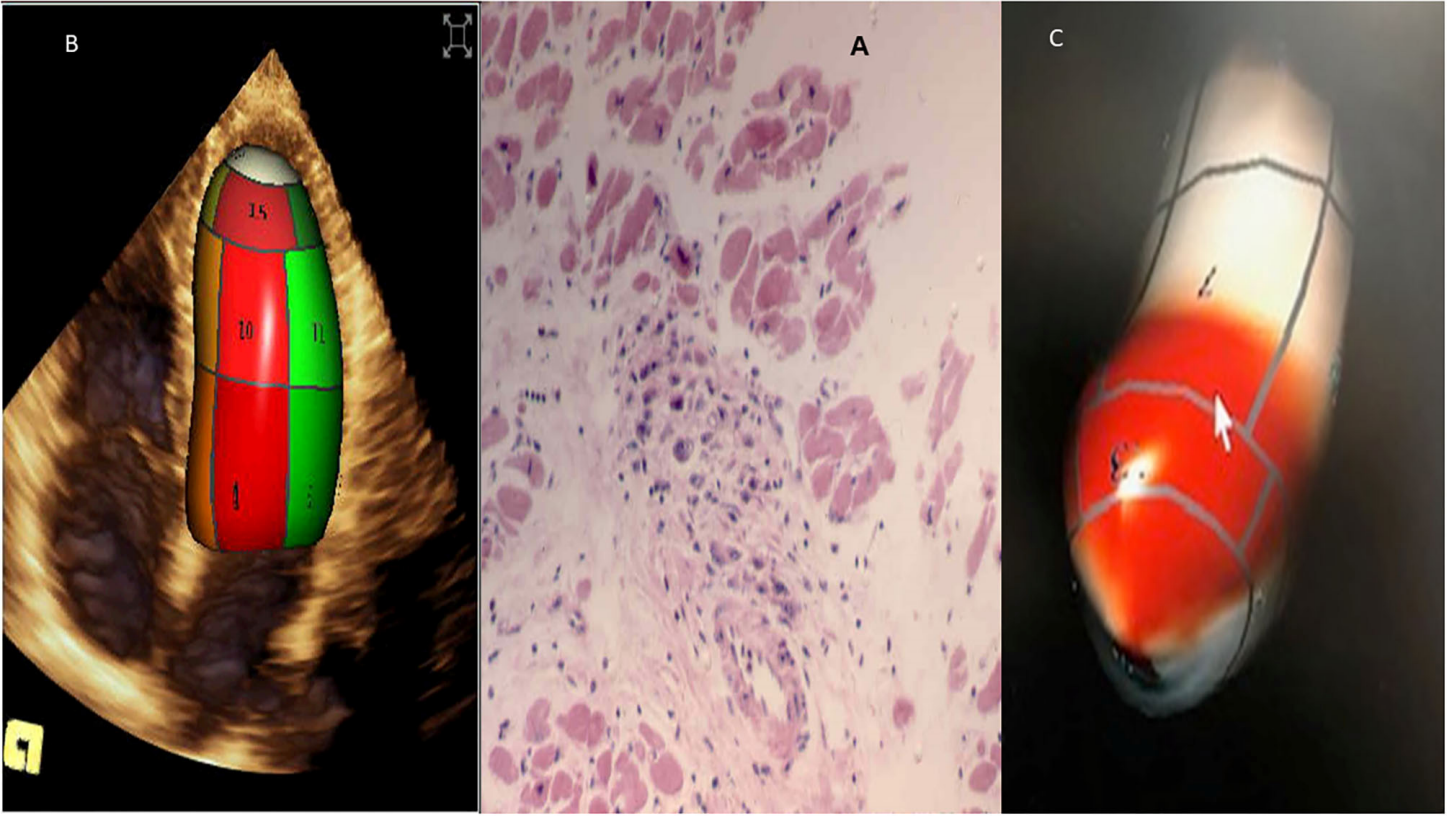
Acute rheumatic fever (carditis). **(A)** Myocardial Aschoff bodies of rheumatic fever (Hematoxylin and eosin stain). **(B)** 3D transthoracic echocardiography depicting normal left ventricle. **(C)** 3D left ventricle presenting myocardial dysfunction, in red abnormal left ventricular synchronicity. 2D Global longitudinal: −12%.

**Figure 5:**
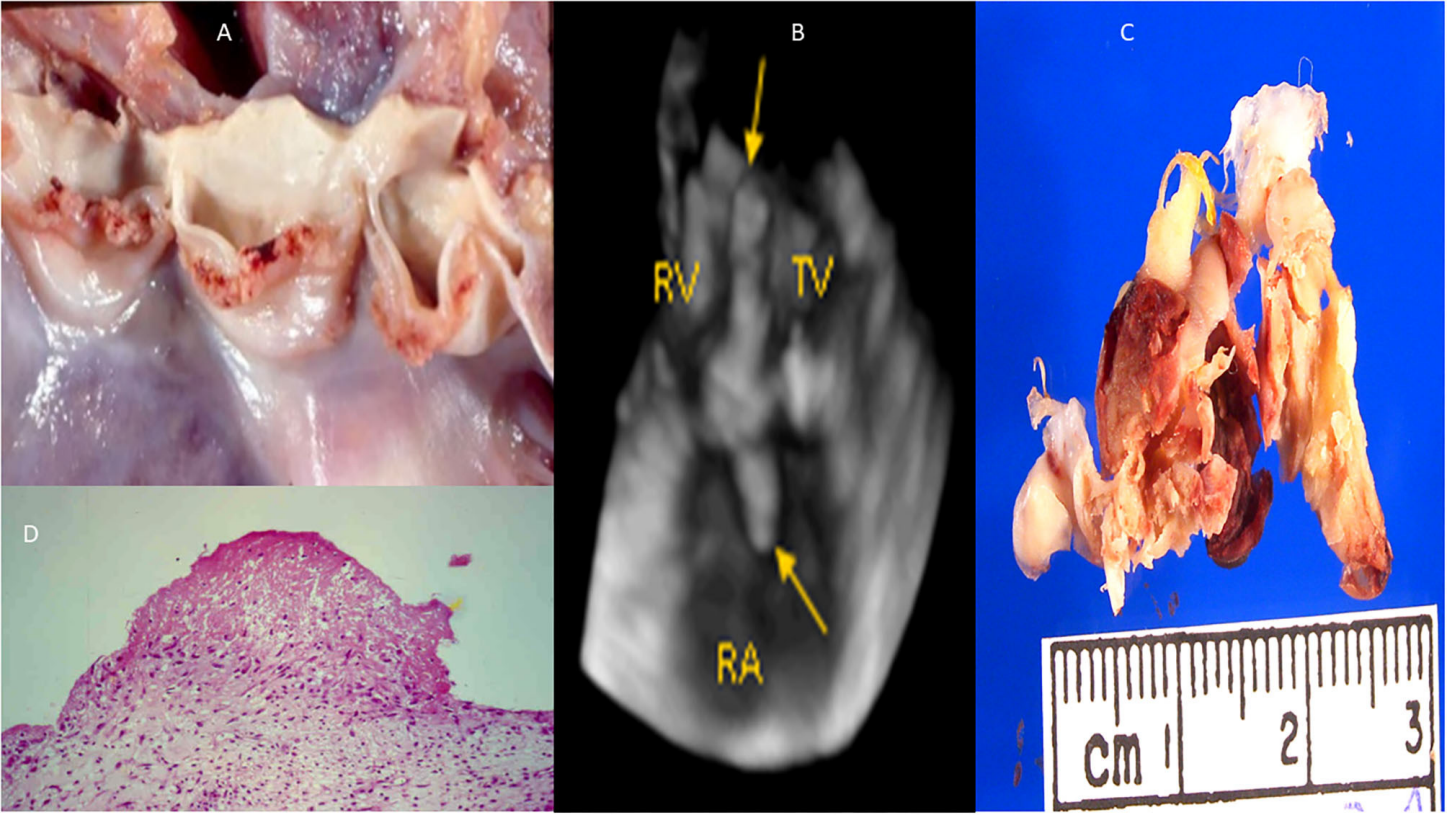
Infective endocarditis (aortic valve and tricuspid valve). **(A)** Macroscopy: small vegetations all over the aortic valve. **(B)** 3D transthoracic echocardiography depicting vegetations over the tricuspid valve (arrows). **(C)** Macroscopy: vegetations all over the tricuspid valve. **(D)** Microcospy: small fibrine thrombi in organization. RV, right ventricle; RA, right atrium; TV, tricuspid valve.

**Figure 6:**
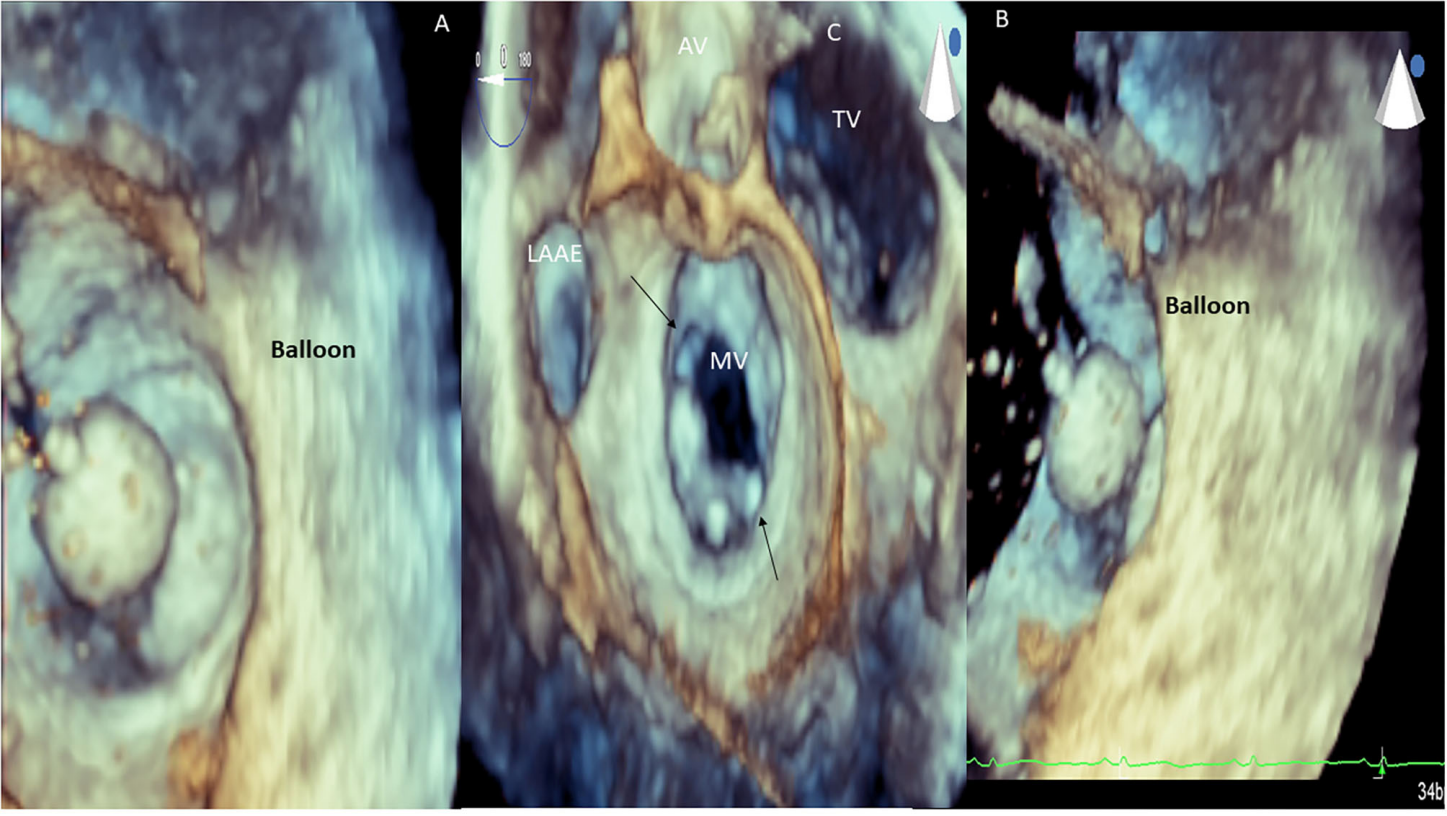
RHD (mitral valve stenosis). 3D transesophageal echocardiography. Demonstration of balloon valvuloplasty in patient with severe mitral valve stenosis **(A,B)**. 3D transesophageal echocardiography. Demonstration of mitral valve commissural opening (arrows, **C**) after balloon valvuloplasty. LAAE, left atrium appendage; AV, aortic valve; MV, mitral valve; TR, tricuspid valve.

**Figure 7:**
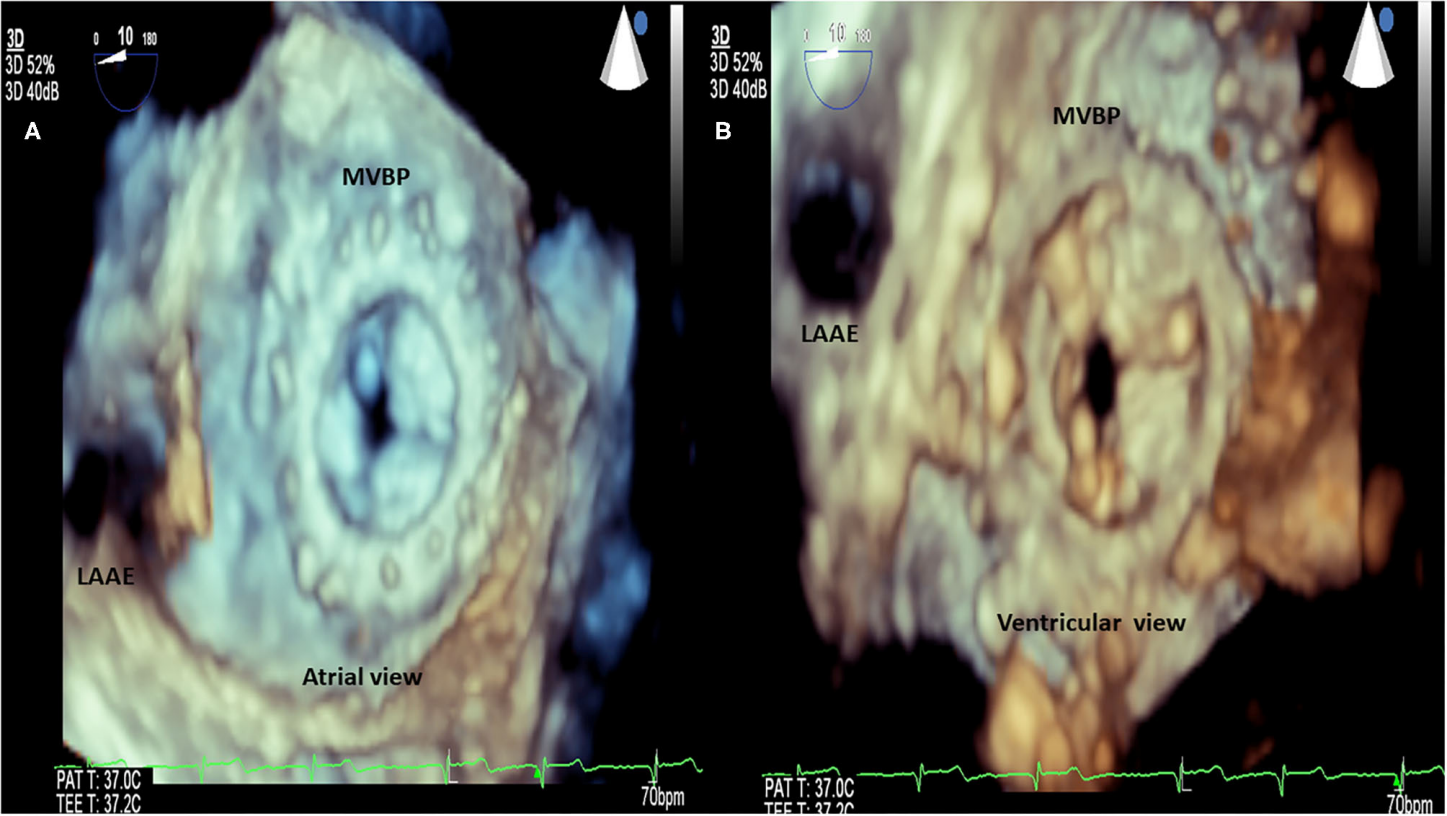
RHD (mitral valve stenosis, stenotic bioprosthesis). 3D transesophageal echocardiography. Demonstration of mitral valve bioprosthesis stenosis. **(A)** Atrial view. **(B)** Ventricular view. LAAE, left atrium appendage; MVBP, mitral valve bioprosthesis.

**Figure 8:**
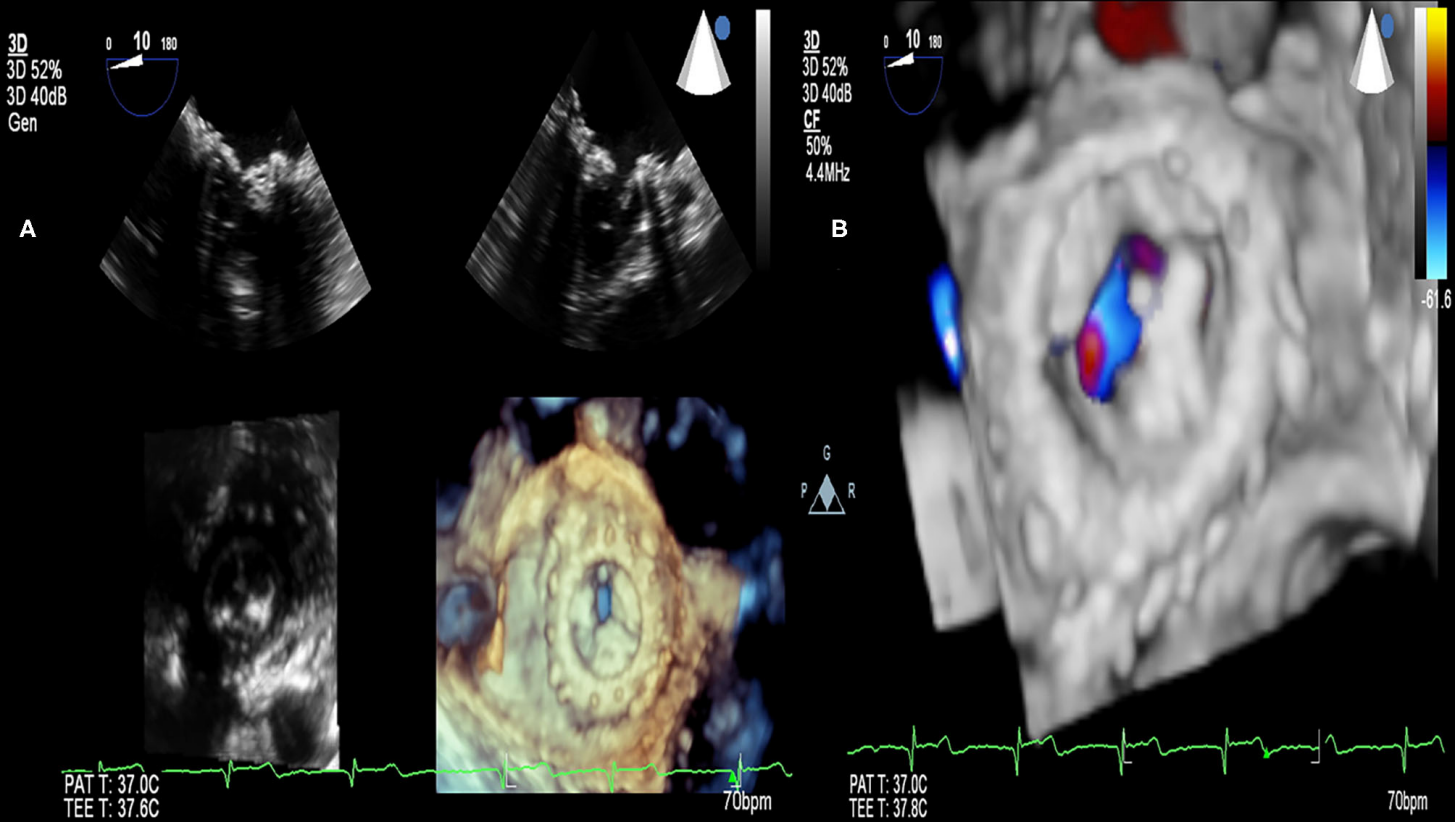
RHD (mitral valve stenosis, stenotic bioprosthesis). **(A)** 3D transesophageal echocardiography. Demonstration of mitral valve bioprosthesis stenosis (comparison with 2D transesophageal echocardiography). **(B)** 3D transesophageal echocardiography. Demonstration of mitral valve bioprosthesis stenosis (color Doppler).

**Figure 9:**
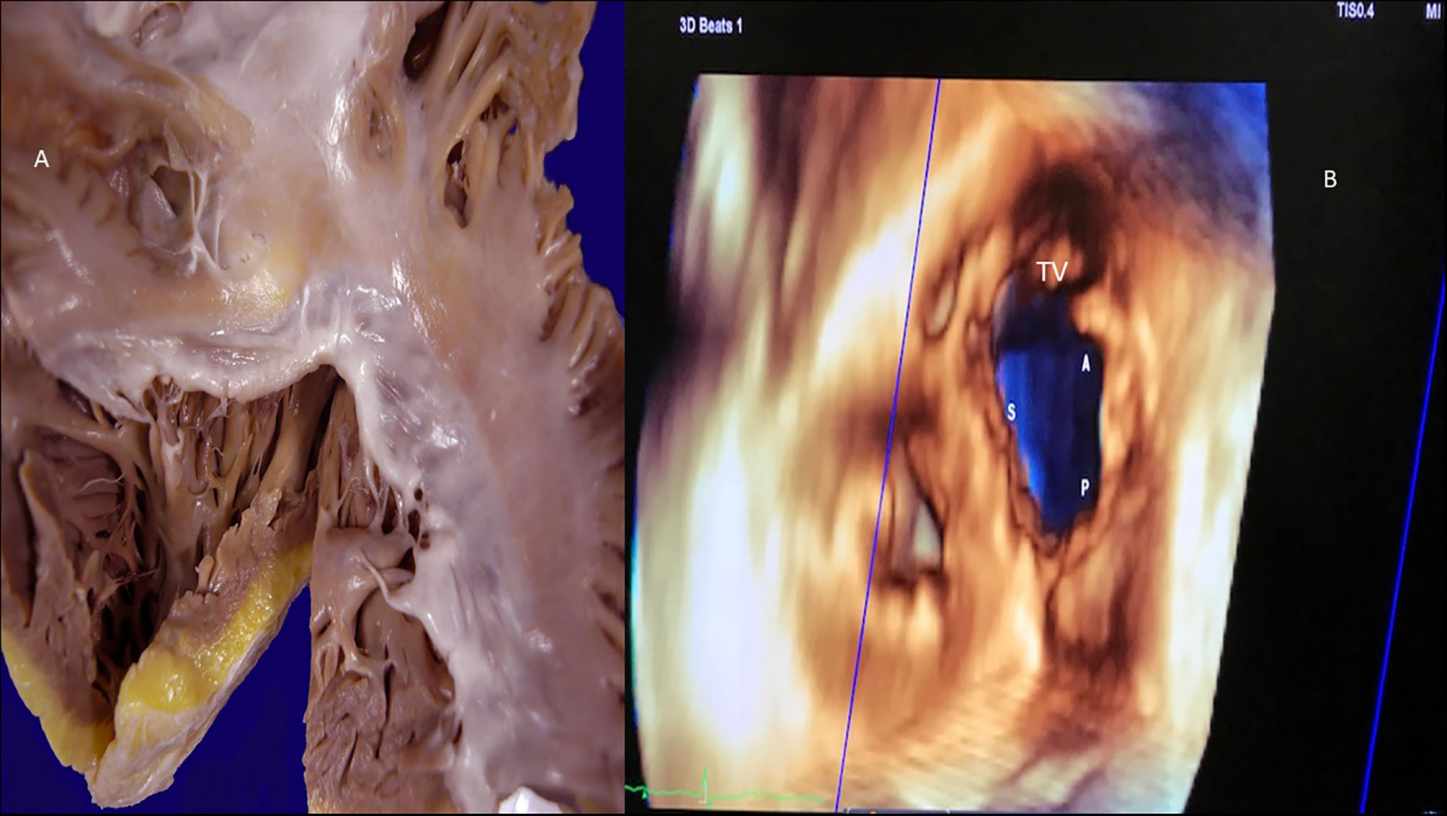
Tricuspid valve rheumatic analysis. **(A)** Macroscopy: the tendinous cords are thickened, fused, and retracted (lack of intercordal spaces), and the anteroseptal commissure is fused. The leaflets show thickening of the free edges. **(B)** 3D echocardiography (right atrial view). Demonstration of lack of coaptation leading to an important regurgitation. S, septal cusp; A, anterior cusp; P, posterior cusp.

**Figure 10:**
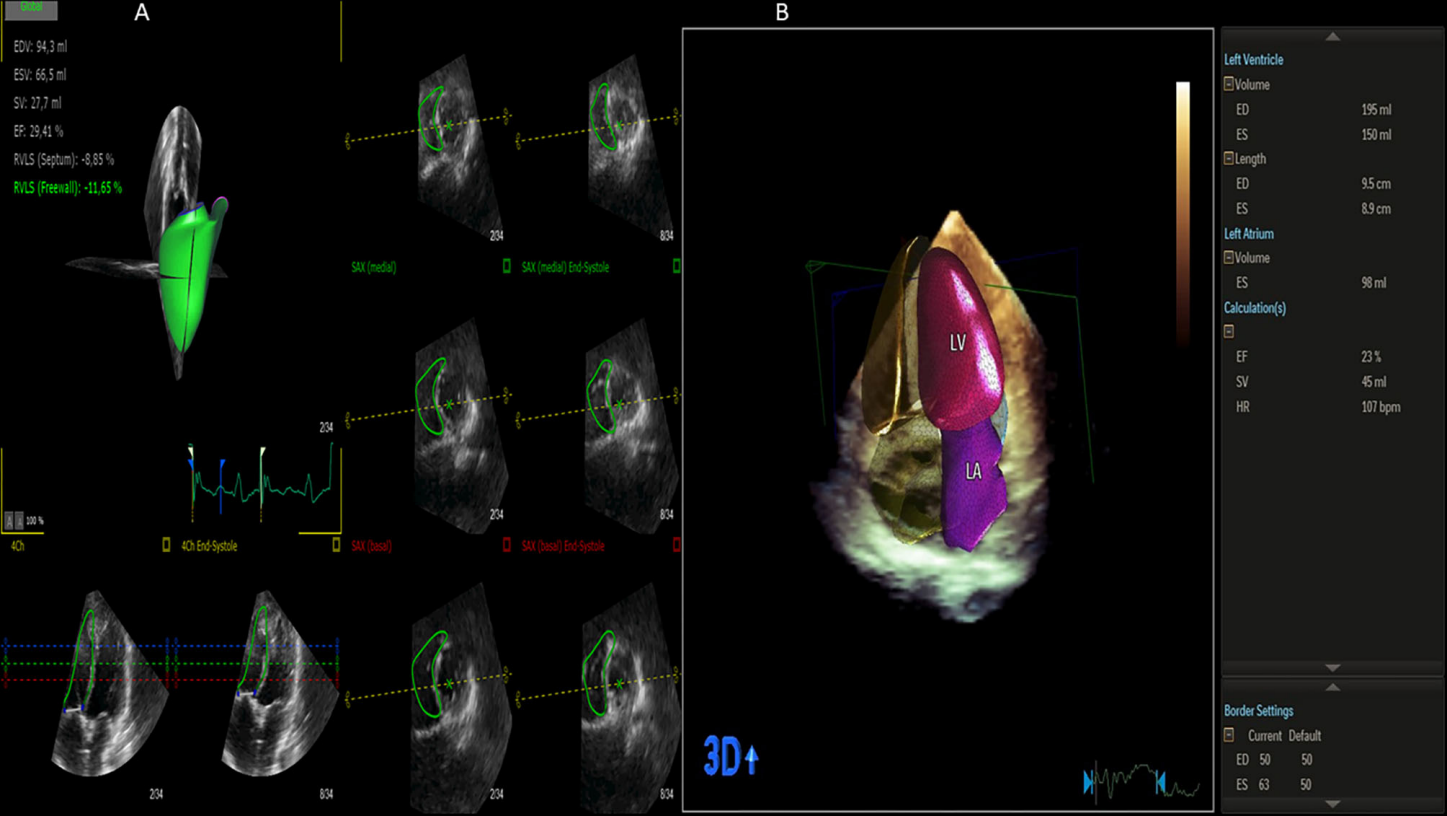
Left and right ventricle analysis by 3D echocardiography (artificial intelligence). **(A)** Right ventricle analysis. **(B)** Left ventricle and left atrium analysis.

**Figure 11:**
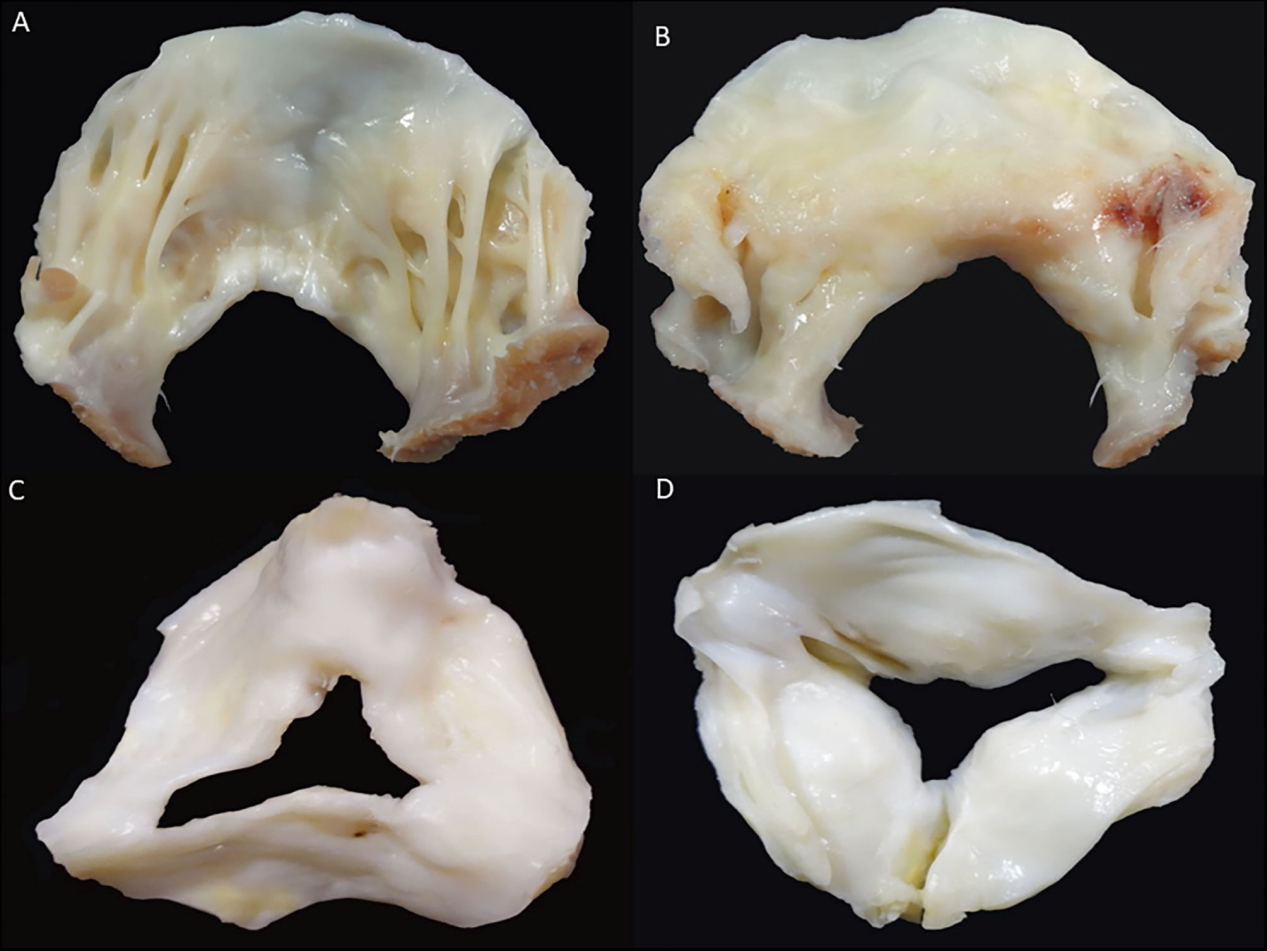
Rheumatic heart disease (Mitral and aortic valves). **(A)** Mitral valve: ventricular surface of the anterior leaflet showing diffuse fibrous thickening. The tendinous cords are thickened, fused, and retracted (lack of intercordal spaces). **(B)** Mitral valve: atrial aspect of the same valvar leaflet showing diffuse thickening and a scar of previous valvular commisurotomy (arrow). **(C)** Aortic valve: surgical specimen showing leaflets thickening and comissural fusion, arterial view. **(D)** Aortic valve: leaflets thickening, commissural fusion and calcification, ventricular view.

**Figure 12:**
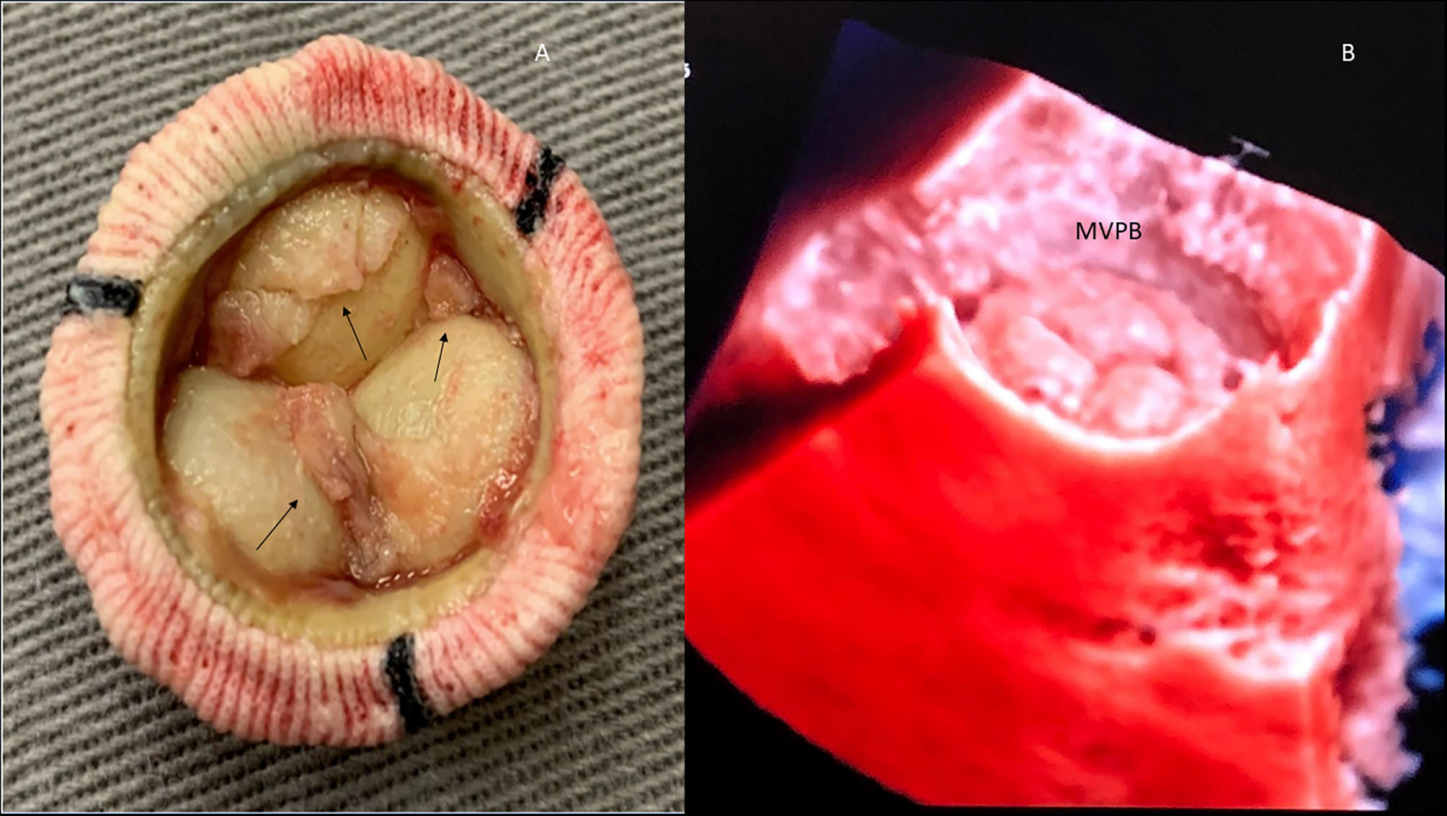
Mitral valve bioprosthesis (MVBP). **(A)** Macroscopy: MVBP in a patient with infective endocarditis. Vegetations (arrows). **(B)** Normal MVBP demonstrated by 3D echocardiography (transluminescence technique).

**Figure 13:**
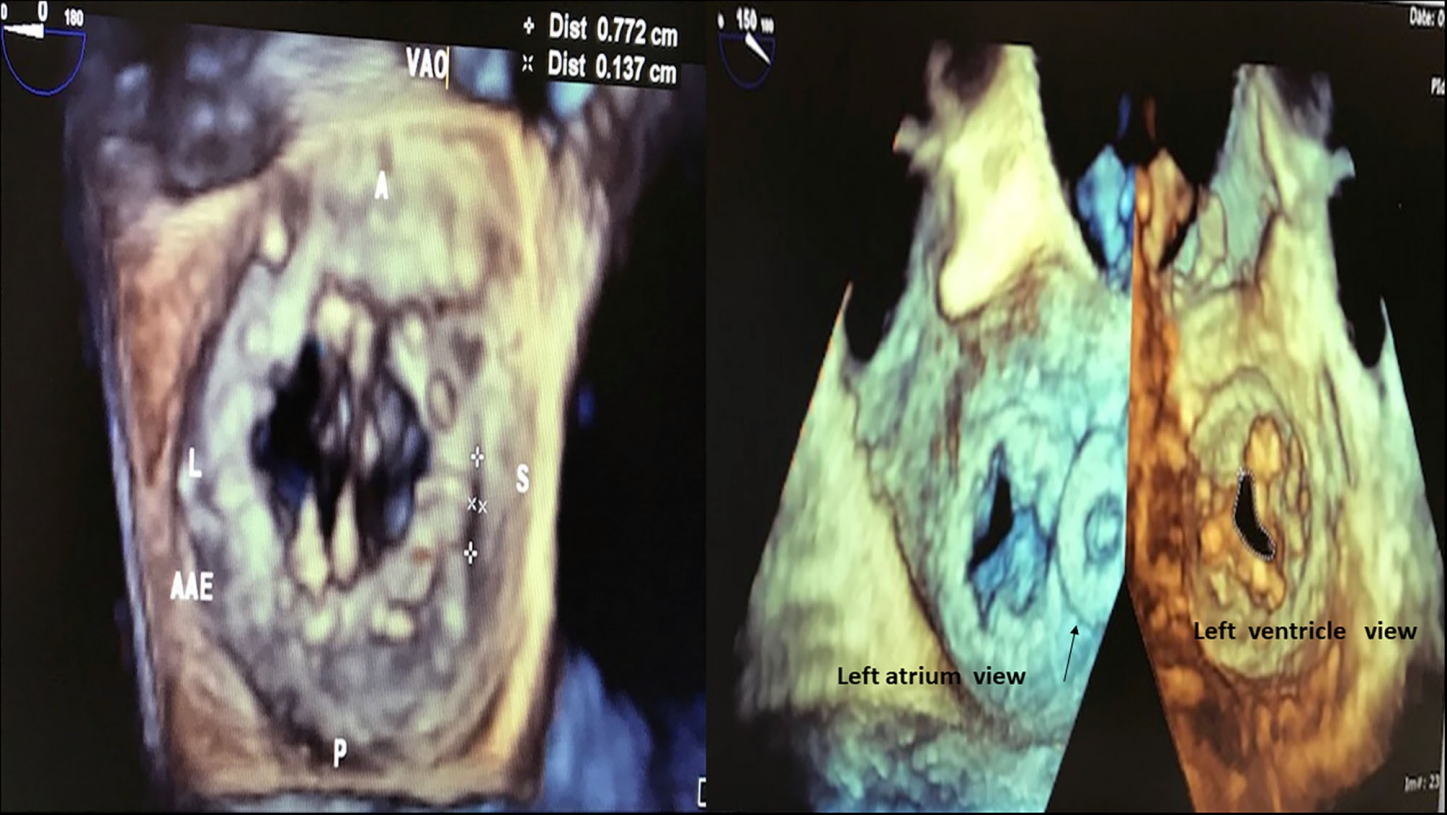
Mitral valve mechanical prosthesis (left) and bioprosthesis (right). Left: Mitral valve mechanical prosthesis presenting periprosthetic leak (7.7 × 1.3 mm) located close to the septal ring. Regions of the prosthetic ring: A, anterior; P, posterior; L, lateral; S, septal; AAE, left atrial appendage; VAO, aortic valve. Right: Mitral valve stenotic bioprosthesis (area: 0.85 cm^2^). Arrow: Amplatzer plug used to treat previous periprosthetic leak.

**Figure 14:**
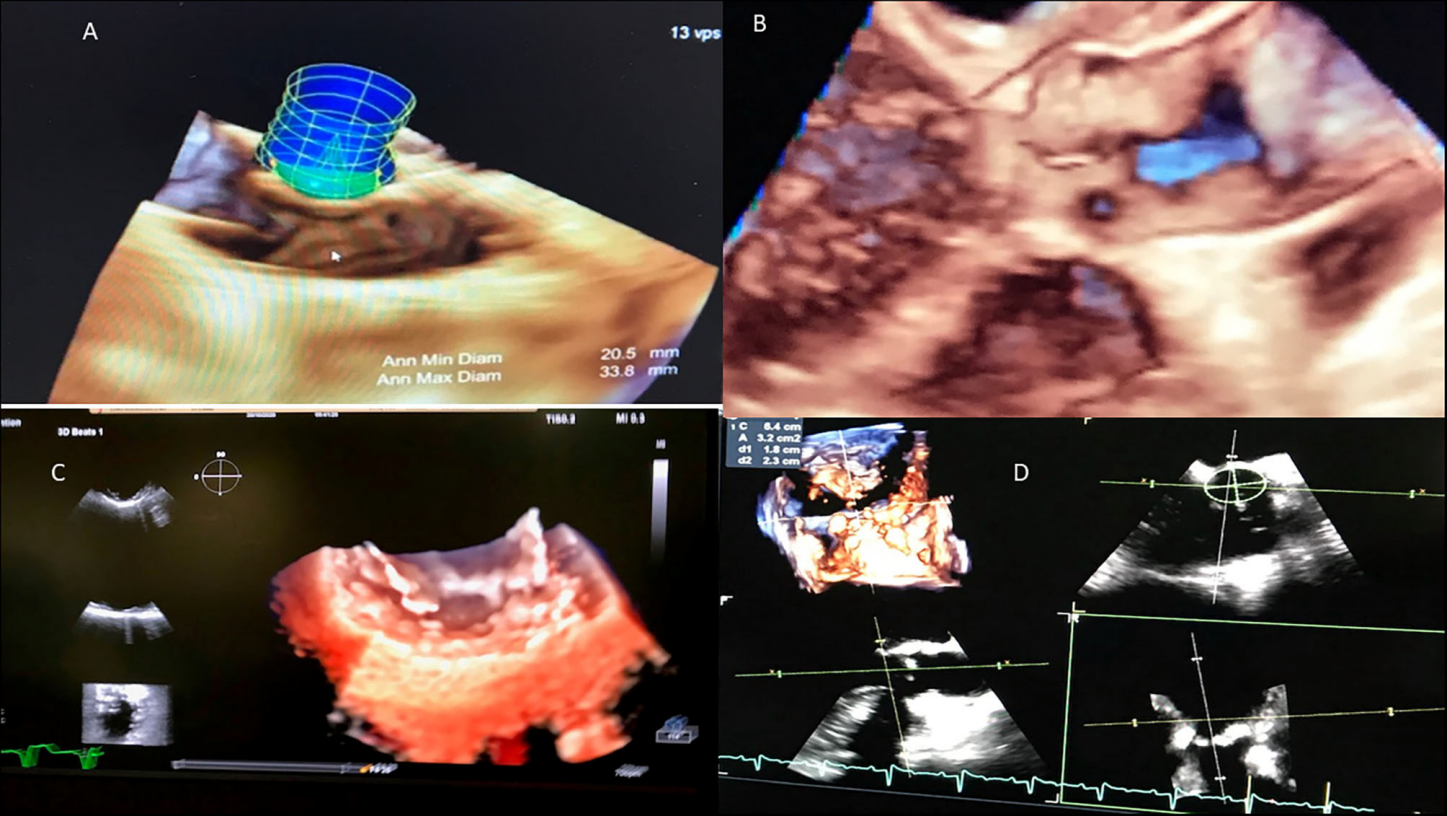
RHD (mitral valve and aortic valve). **(A)** Ventricular surface of the anterior leaflet showing diffuse fibrous thickening. The tendinous cords are thickened, fused, and retracted (lack of intercordal spaces). **(B)** Atrial aspect of the same valvar leaflet showing diffuse thickening and a scar of previous valvular commissurotomy (arrow). **(C)** Surgical specimen showing leaflets thickening and commissural fusion, arterial view. **(D)** Leaflets thickening, commissural fusion and calcification, ventricular view.

**Figure 15:**
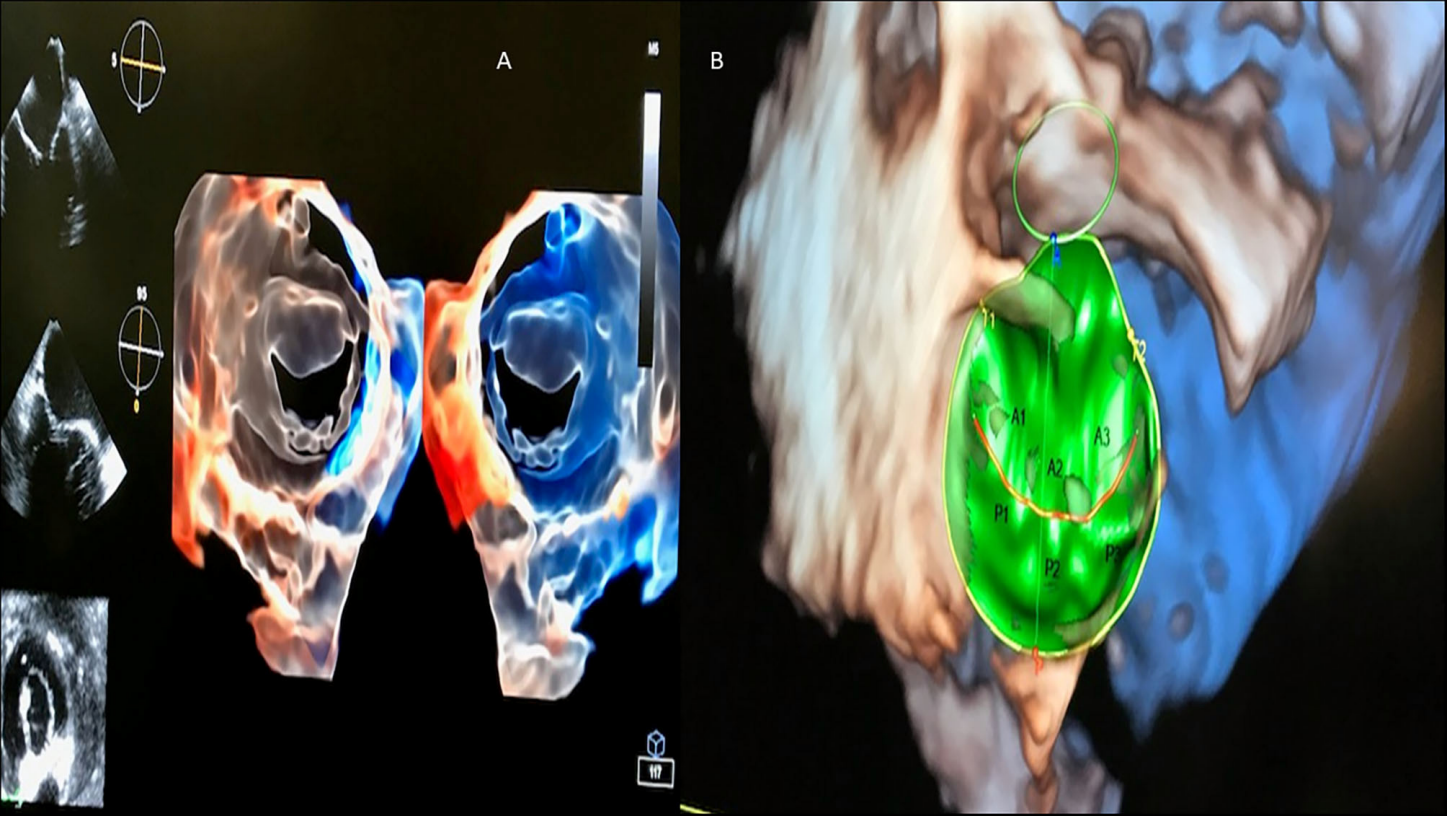
3D transesophageal echocardiography automatic acquisition (in green). **(A)** Mitral valve (transluminescence technique from atrial and ventricular views); **(B)** Mitral valve (surgeon view), depicting valvular segments (A1, A2, A3, P1, P2, P3) from anterior **(A)** and posterior cusps **(B)**.

3D echocardiography results in multi-structural cardiac observation from multiple planes and the rotation of not only the observation planes but also the cardiac structure in relation to a specific investigation plane (13–48). 3D echocardiography is in fact echocardiography in five dimensions, taking into account the three orthogonal planes of structural observation (posteroanterior, lateral medial, and upper inferior), the temporal plane, and the dimension of cardiac flows. It represents an important advance in the anatomical observation of cardiopathies and helps in understanding their pathophysiology and in determining prognostic implications in different clinical situations.

3D echocardiography provides very important clinical applications for the understanding of heart valve diseases, cardiomyopathies, and congenital heart diseases; it is essential for operative planning in cardiac surgeries, for percutaneous transcatheter procedures in the operating room/hybrid room for the treatment of valvular heart disease or congenital heart disease, and for the correction of complications related to surgical treatment of valve diseases (e.g., stenosis of biological prostheses and periprosthetic leaks) (13–48).

3D echocardiography was developed to overcome the limitations of conventional 2D echocardiographic analysis. 3D analysis allows the observation of cardiac structures without the use of mathematical formulae and geometric inferences for cardiac chamber measurement (which is employed during 2D evaluation). 3D echocardiography makes volumetric quantification of the cardiac chambers more realistic and closer to actual anatomical observation. It provides a greater proximity to measurements made by nuclear magnetic resonance (26, 28, 30, 32–34), both from the point of view of cavitary volumes and performance (biventricular ejection fraction and atrial emptying total, passive and active) (31–41), and the measurement of left ventricular mass (30, 38). The use of 3D echocardiography reduces errors in cardiac volumetric measurements, minimizing the foreshortening of the cardiac chambers (which can be a very important limitation concerning 2D echocardiography). The incorporation of three-dimensional echocardiography in cardiac structural analysis also allows cardiac observation from new physiological analysis indices (such as the sphericity and conic index of the left ventricle for prediction of left-ventricle remodeling) and for studying the annular planarity index of the mitral valve ring, as well as the measurement of atrioventricular coupling, concerning dyssynchrony during cardiac resynchronization therapy for heart failure (20, 23, 24).

The idea of 3D echocardiography arises from the reports of Baum and Greenwood during their observations of the human orbit in 1954 (49). In 1974, Dekker et al. demonstrated the possibility of 3D observation of cardiac structures (50). Since then, many researchers, such as Raab and Pearlman, have been involved in the development of 3D echocardiography, even in association with the color Doppler technique by Raqueno and Schott for 3D cardiac structural reconstruction (51, 52). 3D transesophageal echocardiography has also advanced from different investigations in the 80s and 90s, with different groups coordinated by Wollschlager, Pandian, Li, Nandian, Levine, Roelandt, and Picard taking account of different clinical situations (53–57). Currently, we have observed great contributions in real-time 3D echocardiography by investigators such as Lang, Mor-Avi, Badano, Muraru, Kisslo, García Fernández, Peres de isla, and Zamorano (13–16, 21, 22, 33, 42). In this sense, 3D echocardiography seems to be absolutely ready for prime use in RHD and multiple valvulopathy aggression, for a better understanding of cardiac function (biventricular ejection fraction), and to present better anatomical correlation for biventricular remodeling and biatrial enlargement.

## Cardiac Valves

### Rheumatic Lesions and the 3D Echocardiography Approach

#### Mitral Valve Anatomy

The mitral valve apparatus comprises a 3D complex of different structures that enables emptying of the left atrium and filling of the left ventricle during the diastolic phase of the cardiac cycle (58–67). It involves elements such as the mitral valve annulus, leaflets, commissures, chordae tendineae, papillary muscles, and the LV wall with its attached papillary muscle. Any changes in these structures can cause remodeling of the left atrium (mitral stenosis) or the left ventricle (mitral regurgitation), leading to mechanical changes in the left chambers following the onset of RF, and causing modifications over time.

Fully understanding and detailed investigation of the normal mitral valve apparatus is key to better determining the level of RF aggression, imbalance, and distortion.

RHD can affect the mitral valve, causing pure mitral stenosis (25% of all patients) or combined mitral stenosis and regurgitation (40%) (Figure 1). RHD most commonly affects not only the mitral valve but also the aortic or tricuspid valve (multi-valvular aggression) (Figures 9, 14). The disease causes different forms of stenosis of the mitral valve apparatus (commissural, cuspal, chordal, or combined). Thickening of different components of the mitral valve apparatus can occur alone as commissural (30%), cuspal (15%), or chordal (10%), or in combination, and is associated with calcification of the elements. RF aggression most commonly leads to the fusion and thickening of the edges of the cusps and of the chordae. Valvular commissures can be deeply compromised, leading to severe stenosis. The mitral valve ring can also become more rigid due to extension of aggression and from calcium deposits. Therefore, the stenotic mitral valve will have a funnel shape and its orifice can be observed as being a “fish mouth” or “buttonhole.” The degree of calcification is highly related to the increase in transvalvular gradients. When there is less fusion of the valvular commissures and predominant chordae injury, mitral regurgitation is more common. The progression of rheumatic aggression can lead to extensive fibrosis, thickening, and calcification of the entire mitral valve apparatus (Figures 3–5), with even calcification of the wall of the enlarged left atrium.

Two decisive situations that can definitely change not only the mitral valve apparatus anatomy but also the function, and most importantly, impact prognosis, are RF attacks (carditis) (Figure 4) and infective endocarditis. Infective endocarditis can be restricted to the components of the mitral valve apparatus, or not uncommonly, spread to other cardiac valves (Figures 5, 12). Echocardiography is fundamental for the diagnosis of thrombi that is not uncommon in rheumatic patients presenting mitral valve stenosis mainly when atrial fibrillation is observed. However, discrepancy between echocardiographic and histological findings is not unusual, mainly in situations where the differential diagnosis with infective endocarditis is considered.

### Mitral Valve Stenosis

#### 3D Echocardiography

Echocardiography was incorporated into the revised Jones criteria for the identification of rheumatic aggression in 2015 (68), as carditis in the scenario of subclinical carditis or valvulitis (mainly observed as mitral valve insufficiency or aortic valve insufficiency). Mitral valve stenosis occurs when the transmitral mean gradient is higher than 4 mmHg, and there are features related to rheumatic disease aggression. The most characteristic valvular features related to rheumatic aggression are thickening and calcification of the mitral valve apparatus and commissural fusion leading to the restriction of the valvular opening (aspect of “hockey-stick” diastolic opening doming of the valvular cusps). International guidelines recommend that evaluation and analysis of the mitral valve as well as cardiac prosthesis after surgical correction of rheumatic lesions should be undertaken using 3D echocardiography (69, 70). Therefore, the use of 3D echocardiography can provide better spatial analysis for more comprehensive diagnosis and treatment (e.g., for valvuloplasty, native valve replacement, damaged prosthetic valve change, or valve-in-valve procedures) (69, 70) (Figures 8–11).

A score to predict the feasibility and success of mitral valve percutaneous valvuloplasty was originally described by Wilkins et al. in Boston, USA, in 1988, in 22 patients with mitral valve stenosis, with cross-sectional and 2D echocardiography (71). In the 21st century, 3D echocardiography has made it possible to analyze a combination of mitral valve anatomical variables to build a new echocardiographic score for mitral stenosis assessment (72). In this 3D score, with a maximum of 31 points (6 for thickness, 6 for mobility, 10 for calcification, 9 for subvalvular apparatus involvement), mitral valve stenosis can be graded as mild (<8), moderate (8–13), or severe (≥14). It seems that 3D echocardiography can better observe subvalvular involvement (72) and provide commissural observation after valvuloplasty (73–75).

Considering percutaneous balloon mitral valvuloplasty, 3D echocardiography-based scores provided additive information that could predict post-procedural outcome and suboptimal results. 3D echocardiographic analysis enabled a more comprehensive observation of post-procedural posterior-commissural splitting when compared to 2D observation (74).

Although non-invasive multimodality diagnostic investigation has gained tremendous new advances, invasive hemodynamic measurements still present a diagnostic hallmark concerning RHD. The comparison between non-invasive and hemodynamic evaluation is of great concern and interest to cardiac surgeons and clinical cardiologists. For patients presenting mitral valve stenosis, a combination of 3D echocardiography and invasive information (mitral valve navigation system) proved to provide a better correlation to invasive measurement of the mitral valve area measured by the Gorlin equation, when compared to the three-dimensional planimetry method (75).

Another important issue that 3D echocardiography could provide additional information on is related to the analysis of the left atrium (volumes and function) (19, 76, 77). For instance, after balloon valvuloplasty, improvements in left atrial reverse remodeling (decreased volumes) and left atrial emptying fraction (increased) were observed 72 h and 12 months after the procedure (76). Patients presenting with large left atria can have very low emptying fractions leading to irregular rhythms, similar to atrial fibrillation. In addition, 3D echocardiography could provide important prognostic information concerning the shape of the left atrium and the likelihood of embolic cerebrovascular events in mitral stenosis (77). It was observed that a more spherical LA shape, as determined by the use of 3D echocardiography, was associated with an increased risk of embolic cerebrovascular events (77). The mitral valve area can be analyzed by echocardiography using different analyses: two-dimensional planimetry, continuity equation, pressure half time (PHT) technique, proximal isovelocity approach (PISA), and three-dimensional evaluation. 3D echocardiographic analysis enables multiplane guidance for mitral valve evaluation, providing an accurate measurement of the mitral valve area, and overcoming the sources of errors of other echocardiographic techniques without employing mathematical equations or calculus.

3D echocardiography can also provide automatic information concerning the left atrium appendage, when considered to be percutaneously closed.

### Mitral Valve Insufficiency

#### 3D Echocardiography

Mitral valve rheumatic disease regurgitation caused by rheumatic valvular lesions is observed as thickening, calcification, scarring of the cusps, chordal shortening leading to valvular lack of coaptation, and restriction of valvular movement. Complete mitral valve rheumatic disease regurgitation analysis should employ different echocardiographic techniques for complete anatomical and morphological investigation (to differentiate organic from functional mitral valve insufficiency) and to grade the insufficiency. Thus, we should try to obtain parameters such as vena contracta, vena contracta area, regurgitant volume, regurgitant fraction, and regurgitant orifice.

3D echocardiography enables multi-angular spatial observations, mainly in the very particular “en face” view of the mitral valve. It also allows not only the ventricular view but also the ventricular approach, as well as simultaneous observations from the left atrial and ventricular views. 3D transesophageal observation enables a perfect analysis, as in the surgical view from the operating room. In addition, 3D color flow echocardiography enables accurate identification of the origin of the regurgitant jet and the quantification of the number of jets. The quantification of 3D mitral regurgitation vena contracta is employed as a semiquantitative method to grade insufficiency and can be used to overcome two-dimensional PISA limitations (64, 78, 79). 3D echocardiographic analysis of mitral insufficiency is closely correlated to magnetic resonance evaluation (80) and could be employed to predict mitral valve surgical repair results (81). The measurement of the effective regurgitant orifice area (ERO) with the 2D PISA technique underestimates the values of the vena contracta area obtained by the 3D PISA technique, the more elliptic or more asymmetric it is (78, 82).

3D echocardiographic analysis of the mitral valve is very important and valued for transcatheter treatment of mitral disease while managing periprosthetic leaks and valve-in-valve procedures.

In acute attacks of RF, the presence of small vegetations on the line of closure of the insufficient mitral valve may lead to a misleading gross and echocardiographic diagnosis of infective endocarditis, even more because the acute RF attack is a febrile disease.

Also, 3D echocardiography can be currently employed to provide automatic information from the mitral valve apparatus, concerning the annulus dimensions, annulus height, tenting area, planar and non-planar angles, shape, sphericity index, intertrigonal and intercommissural distances, and information from the leaflet coaptation (area, width).

### Aortic Valve Anatomy

Similar to the mitral valve, echocardiographic analysis of the aortic valve requires detailed knowledge of the aortic valve and aortic root anatomy, including the anatomical complex of the aortic root. It comprises the sinuses of Valsalva, the valve leaflets, and the fibrous triangular inter-leaflets. 3D echocardiography allows direct and real-time visualization of all components of the aortic valvar complex, ensuring excellent anatomical proximity (83, 84). The use of 3D echocardiography allows for accurate measurements of the height of the coronary ostia and the diameters of the aortic root (aortic annulus, sinuses of Valsalva, and sinotubular junction), which are important parameters for percutaneous procedures such as percutaneous implantation of aortic prosthesis (TAVR) (84). An excellent correlation was observed between the diameters obtained by 3D echocardiography and those derived from aortic angiotomographic analysis. Aortic valve insufficiency (47%) is more common than aortic stenosis (14%) in patients with rheumatic heart disease (85). The aortic valve area, assessed by echocardiography, is 4.0 ± 0.8 cm^2^. On analysis, measurement with 3D echocardiography provides greater accuracy, showing better reproducibility. 3D echocardiographic analysis also allows better observation of the etiology of aortic valve disease, allowing for a better structural observation of anatomical changes such as rheumatic disease lesions, biscuspid valves, quadricuspid valves, degenerative changes such as Lambl's excretions, and tumors such as papillary fibroelastomas (86). Multiplanar 3D transesophageal analysis of the rheumatic aortic valve can undoubtedly add anatomical information for imaging diagnosis.

### Aortic Valve Stenosis

#### 3D Echocardiography

Rheumatic aortic valve disease can lead to commissural fusion, fibrosis, thickening and calcification of the leaflets, retraction of the leaflet edges, and turning of the systolic aortic orifice into a more rounded or triangular shape. The assessment of the severity and relevance of aortic stenosis must take into account anatomical and functional aspects. Thus, it is important to analyze the aortic valve morphology, the presence and distribution of calcium along the valve commissures and leaflets, and the central or peripheral distribution of calcium. We must also obtain the valve area as well as the maximum and medium transvalvular gradients, the maximum speed of the left ventricular outflow tract, the contractile performance of the left ventricle (ejection fraction and myocardial deformation), the degree of left ventricular hypertrophy, analysis of left ventricular diastolic function, the analysis of arterial valve impedance (especially in hypertensive patients), the analysis of associated heart valve diseases (mitral and tricuspid regurgitation), the degree of hypertension, and the analysis of the performance of the right ventricle.

Analysis of the aortic valve area is of great importance in analyzing the severity of aortic stenosis. A widely used method for measuring the aortic valve area is the continuity equation using 2D echocardiography. In this method, a circular shape is assumed for the ventricular outflow tract, which, in a large number of cases, is different from the actual elliptical anatomical shape (87). This inference can cause an underestimation of the aortic valve area. Planimetry of the left ventricular outflow tract using 3D echocardiography allows for greater accuracy in the assessment of the aortic valve area from an en-face perspective (42). 3D planimetry of the aortic valve area provides accurate results, even when compared to invasive measurements of the aortic valve area, with several studies documenting superiority over the 2D method (42, 88).

Another method for assessing the aortic valve area consists of using the left ventricular stroke volume measured by 3D analysis (42). To measure the aortic valve area, stroke volume is divided by the aortic valve time–velocity integral (42). The accuracy of this method has proven to be adequate and even superior to measurements performed using the continuity equation and other 2D volumetric methods.

Measurement of the aortic area using the 3D LV stroke volume method is as follows:

1. Left-ventricle stroke volume, measured by 3D echocardiography
2. Aortic valve velocity time integral (TVI)

### Aortic Valve Area

#### Left-Ventricle Stroke Volume (cm^3^)

##### TVI (Aortic Valve) (cm)

This method, using left-ventricle stroke volume for the evaluation of the aortic area, has better correlation with invasive methods (Gorlin equation) than bidimensional methods.

The analysis of the severity of aortic stenosis using 3D echocardiography has better correlation with measurements using other imaging methods (computed tomography and magnetic resonance imaging), when compared with 2D echocardiographic analysis (89). In addition, left ventricular mass analyzed by 3D echocardiography is better correlated with magnetic resonance evaluation than 2D echocardiographic evaluation. The use of 3D echocardiography can lead to reclassification of aortic valve stenosis severity in 10–25% of cases (90).

### Aortic Valve Insufficiency

#### 3D Echocardiography

The use of echocardiography for quantification and evaluation of the hemodynamic and anatomical impact of aortic valve insufficiency allows accurate observation and determination of the temporal moment of the natural history of patients with aortic insufficiency. The morphofunctional analysis of the patient with aortic insufficiency should take into account the etiological evaluation of the valve disease, the diameters of the ascending aorta, the diameters of the left ventricle, myocardial performance analysis (left ventricular ejection fraction), the quantification of severity of insufficiency with measurement of the contracted vena, the observation of the width of the jet in the left ventricular outflow tract, the measurement of the regurgitant fraction, the regurgitant volume, and the ERO.

The use of 3D echocardiography provided new insights into the analysis of the severity of aortic valve insufficiency (70, 89, 91). Apart from the anatomical approach, 3D echocardiography has also enabled better understanding of the bidimensional vena contracta measurement (2D color echocardiography could lead to inaccurate evaluation and incorrect geometric inferences for the analysis of the regurgitant orifice, which, while initially thought to be plane and circular, has in many cases proved to be elliptical) (91). Thus, the use of a 3D approach increases the accuracy of analysis of the aortic valve regurgitant lesion, enabling the direct measurement of the vena contracta area of the regurgitant jet. Furthermore, for transcatheter aortic valve approaches in rheumatic patients who have undergone previous surgical correction and who present with complications such as periprosthetic leak, the use of 3D echocardiography is of utmost importance to guide transcatheter procedures (plug implantation).

### Tricuspid Valve Stenosis and Regurgitation

#### 3D Echocardiography

Isolated tricuspid aggression in RHD is rare and is most commonly associated with mitral and aortic disease. The normal tricuspid area varies from 4 to 6 cm^2^. Tricuspid rheumatic valve disease demonstrates similar findings to those observed in mitral valve aggression. Thus, fusion and thickening of the cusps, diastolic cuspal doming, thickening, shortening, fusion, and retraction of the chordae, commissural thickening and fibrosis, and calcification/scarring of the different elements of the valvular apparatus can be found over time after rheumatic onset (Figure 12). These valvular alterations can lead to stenosis, insufficiency, or combined tricuspid lesions (more common). As in other rheumatic valvular diseases, tricuspid disorders should be analyzed considering anatomical and morphofunctional features. Tricuspid valve stenosis is considered to be severe when the tricuspid area is <1.0 cm^2^; the diastolic mean transvalvular gradient is ≥5 mmHg when the tricuspid PHT is ≥190 ms; and inflow velocity integral is ≥60 cm, with consequent right atrial enlargement. The use of 3D echocardiography can lead to a better understanding of tricuspid rheumatic anatomy and derived atrium changes, right ventricular functional and geometric modifications, and evidence of thrombi in the right atrium (92–97). As for left atrium analysis and remodeling comprehension in different clinical scenarios, a global analysis of the right chambers and tricuspid valve elements is better when done using 3D echocardiography (92–97). The tricuspid area can be analyzed from an atrial or ventricular perspective, enabling an en-face wider view of the valve.

This en-face view provides a simultaneous view of the three leaflets, leading to the measurement of the vena contracta area of the tricuspid regurgitant jet.

A 3D vena contracta area cutoff value of 0.61 cm^2^ can determine severe tricuspid regurgitation, sensitivity of 78%, and specificity of 97% (97). The measurement of ERO with the 2D PISA technique underestimates the values of the vena contracta area obtained by the 3D PISA technique, in particular for eccentric jets (97).

Another important piece of information derived from 3D echocardiography is the evaluation of right-ventricle performance (3D right ventricle volumes and ejection fraction).

Another important condition that can be very well-visualized with the addition of 3D echocardiography is tricuspid lesions due to infective endocarditis (Figure 13).

### Pulmonic Valve Stenosis and Regurgitation

#### 3D Echocardiography

Rarely is isolated pulmonic valve aggression observed after RHD establishment. Whenever present, it is more commonly associated with mitral valve stenosis (98). The pulmonic valve is considered to have severe stenosis when the pulmonic valvular area is <1.0 cm^2^ in adults, with a peak transpulmonic flow velocity of >4 m/s, along with the demonstration of restriction of leaflet mobility with a doming appearance on systole, calcification, and thickening of the leaflets. The use of 3D transesophageal echocardiography may add diagnostic information to anatomical investigation of the pulmonic valve, when compared to the 2D echocardiographic approach (99).

### Mixed Rheumatic Valve Disease

#### 3D Echocardiography

Mixed rheumatic valvular disease is common during the follow-up of patients with rheumatic valvular disease (100). Multiple valvular disease is considered whenever two or more valvular disorders are present and the functional valve disease is secondary to primary organic valvular involvement. In this sense, it is important for both anatomical evaluation and morphofunctional multivalvular occurrence, considering valvular lesions as well as chamber remodeling (atria and ventricle).

In this sense, the use of 3D echocardiography in valvular heart disease will provide valvular anatomical information as well as biventricular functional analysis (ejection fraction), atria volumes, and emptying fractions.

## Limitations

3D echocardiography techniques are subject to the physical limitations of ultrasound, such as inadequate image quality, planar variation within the acquired image, the occurrence of image artifacts, gain adjustments (such as considering the dependence of valvular flow orifice sizes), the occurrence of cardiac arrhythmias, and breathing variations during image acquisition. Also, the possible occurrence of artificial thickening of the cardiac structures displayed in volume rendering, the unreliability for tissue characterization, and the interobserver bias (which is considered to be decreased with the use of automatic quantification and artificial intelligence) are of great importance, in addition to the need for specific training to obtain and analyze the images. In developing countries, 3D echocardiography equipment is still limited by cost. In the future, a better analysis of RHD patients should be considered, taking into consideration a multimodality approach (considering 3D echocardiography, computed tomography, and cardiac magnetic resonance).

## Conclusion

RHD is a very important health issue worldwide, mainly in underdeveloped countries. Echocardiography is the most relevant imaging technique providing diagnostic information, enabling prognostic data, and presenting a very important role in the correction of complications after surgical repair of rheumatic heart valvulopathies. Accordingly, 3D echocardiography is ready for routine use in patients with RHD presenting with valvular abnormalities.

## Author Contributions

MV, CB, AG, PV, LB, LD, PG, VA, FT, and RS: planning, conduct, and reporting. MV: guarantor. All authors contributed to the article and approved the submitted version.

## Conflict of Interest

The authors declare that the research was conducted in the absence of any commercial or financial relationships that could be construed as a potential conflict of interest.
